# Supplementary material for: An Evaluation of Medication Prescribing Patterns for Acute Migraine in the Emergency Department: A Scoping Review
Source: J Clin Med. 2021 Mar 12;10(6):1191. doi: 10.3390/jcm10061191 (PMC7998873; doi:10.3390/jcm10061191)
Supplement: Supplementary file 1 [file jcm-10-01191-s001.pdf]

Supplemental material S1: Description of the manual search strategy

The aforoementioned keywords(Migraine, acute care, emergency department, analgesic, triptan, opioid and NSAID) were searched using google scholar with a custom range of 2000 until 2020. Search results were sorted by relevance and titles on the first three pages of results were screened for relevance. The manual search did not contribute to eligible papers for inclusion in our scoping review.

Table S1: Search strategy example from Ovid Medline

#	Searches	Results
1	exp Migraine Disorders/	27361
2	migraine*.ti,ab.	34929
3	1 or 2	39583
4	exp Emergency Service, Hospital/	79345
5	((emergency or trauma or acute) adj2 (department* or service* or unit* or centre* or center* or care)).ti,ab.	169642
6	4 or 5	197348
7	exp Analgesics/	534101
8	exp Tryptamines/	93416
9	exp Analgesics, Opioid/	116848
10	exp Anti-inflammatory Agents, Non-steroidal/	198161
11	(analgesic* or triptan* or opioid* or NSAID*).ti,ab.	177757
12	7 or 8 or 9 or 10 or 11	701733
13	3 and 6 and 12	305

Table S2: Search strategy example from Ovid Medline

**Database**	**Returned results**
Ovid MEDLINE(R) ALL, Embase Classic + embase, APA Psycinfo	1008
Pubmed	131
Cochrane	60
CINAHL	86
PsycInfo	17
**Total**	1302

The searches were performed between the 5th of September 2020 and the 24th of September 2020. The following table shows the number of studies attained from each search.

Table S3: Rationale for exclusion with respective number of articles excluded.

**Rationale for exclusion**	**Number of articles excluded**
Inappropriate outcomes	156
Inappropriate design	45
Inappropriate population	56
Outside inclusion criteria	4
Unfiltered duplication	6
**Total**	267
